# Distribution of Choroidal Thinning in High Myopia, Diabetes Mellitus, and Aging: A Swept-Source OCT Study

**DOI:** 10.1155/2019/3567813

**Published:** 2019-08-15

**Authors:** Francisco de Asís Bartol-Puyal, Carlos Isanta, Óscar Ruiz-Moreno, Beatriz Abadia, Pilar Calvo, Luis Pablo

**Affiliations:** ^1^Ophthalmology Department, Miguel Servet University Hospital, Zaragoza, Spain; ^2^Ophthalmology Innovative and Research Group (GIMSO), Aragón Institute for Health Research (IIS Aragón), Zaragoza, Spain; ^3^University of Zaragoza, Zaragoza, Spain

## Abstract

**Purpose:**

To compare the macular choroidal thinning between young healthy, aged healthy, young high myopic, and aged type 2 diabetic (T2D) patients using the Early Treatment Diabetic Retinopathy Study (ETDRS) grid and three-dimensional (3D) maps.

**Methods:**

A prospective study including 102 eyes of 51 healthy young subjects, 60 eyes of 30 healthy aged subjects, 24 eyes of 12 high myopic patients, and 110 eyes of 55 T2D patients. Choroidal thickness (CT) was examined with swept-source optical coherence tomography Triton DRI (Topcon Corporation, Tokyo, Japan). The choroid was automatically segmented using the software algorithm, and mean CT values of a 6 × 6 mm macular cube were exported. 3D maps were created to represent CT, and its values were compared using the ETDRS grid.

**Results:**

Mean age was 27.31 ± 3.95, 66.41 ± 7.54, 27.69 ± 3.89, and 66.48 ± 7.59 years in young healthy, aged healthy, young high myopic, and T2D patients, respectively. CT was not shown to be uniform, as superior and central zones were thicker. All ETDRS sectors were always thicker (*p* < 0.05) in young healthy individuals than in the others. It was found that the choroidal sector which got thinner was inferior in case of age (103.28 *μ*m decrease), inferior-nasal in high myopia (86.19 *μ*m decrease), and temporal in T2D (55.57 *μ*m decrease). In addition, the choroid got thinner in those regions where it was thicker in healthy subjects.

**Conclusions:**

3D maps allow a further comprehension of choroidal changes. The choroidal pattern in young healthy individuals resembles a mountain range; with age, a mountain peak; in high myopia, an inverted gorge; and in aged T2D, gathered hills. Not all choroidal regions are affected in a similar way, as it depends on the pathology. The thicker the zone is in healthy subjects, the thinner it becomes with any pathology.

## 1. Introduction

With the advent of optical coherence tomography (OCT) technology, the choroid has been precisely visualized for the past few years. It has been proved to play an important role in different retinal disorders such as myopia, central serous chorioretinopathy, and age-related macular degeneration. Quantitative assessment of the choroid has allowed new research findings to differentiate normal from pathological processes within the choroid. It is known that choroidal thickness (CT) varies with age [1–3], axial length (AL) [4–7], day time [8–10], and race [11]. A choroidal thinning has been found in pathologies such as myopia [12] and diabetes mellitus [13–15], and a relevant thickening has been found in the pachychoroid spectrum, which includes the polypoidal choroidal vasculopathy [16].

Swept-source OCT (SS-OCT) is the last generation of OCT, and it uses a laser source of a longer wavelength (1050 nm) which penetrates deeper in the retinal and choroidal tissues than conventional laser sources used in previous spectral-domain OCT devices [17].

SS-OCT provides retinal and choroidal macular thickness geographically displayed as a false-color topographic map, and it is numerically reported as averages in each of the nine regions defined by the Early Treatment Diabetic Retinopathy Study (ETDRS) [11]. The ETDRS grid includes a central disc of 500 *μ*m of diameter (foveal region) and an inner and an outer ring; each one was divided into four quadrants, with a diameter of 3000 *μ*m and 6000 *μ*m, respectively. This grid is used for quantitative evaluations of either retinal or choroidal thickness.

This study aimed to evaluate the distribution of choroidal thinning in high myopia, diabetes mellitus, and aging using the classic ETDRS grid from SS-OCT and a new different mapping.

## 2. Methods

### 2.1. Sample Selection

Over a 2-year duration (from November 2015 to November 2017), we performed a cross-sectional SS-OCT study on four different groups of patients: young healthy subjects (group 1), senior healthy subjects (group 2), young high-myopic patients (group 3), and patients with type 2 diabetes mellitus (T2D) (group 4). All patients underwent a complete ophthalmic evaluation at the Miguel Servet University Hospital in Zaragoza, Spain. The study protocol adhered to the tenets of the Declaration of Helsinki and was approved by the Institutional Review Board (Clinical Research Ethics Committee of Aragón (CEICA)).

Exclusion criteria were a race different from Caucasian, any ocular pathology or previous treatment, amblyopia, endocrine or neurological diseases, cancer history, corticosteroids, and immunosuppressive drugs.

Group 1 included young healthy volunteers between 18 and 35 years old and with an AL ≤ 25 mm. Group 2 included senior healthy volunteers between 55 and 75 years old and with an AL ≤ 25 mm. Group 3 included young healthy patients between 18 and 35 years old but with an AL ≥ 25 mm. Group 4 included T2D patients between 55 and 75 years old, with mild or moderate diabetic retinopathy (DR), without macular oedema and without any previous ophthalmological treatment. Healthy individuals' medical records were examined in order to verify that all of them had no systemic illnesses. T2D patients were diagnosed after the criteria of the American Diabetes Association, and all of them were negative for anti-glutamic acid decarboxylase antibodies.

### 2.2. Study Protocol

Patients underwent a deep ophthalmological examination which included best-corrected visual acuity (BCVA), refraction, slit-lamp examination, intraocular pressure (IOP) with Goldmann applanation tonometry, optical biometry (IOLMaster 500, Carl Zeiss Meditec, Jena, Germany), indirect funduscopy, and SS-OCT Triton Deep Range Image (Topcon Corporation, Tokyo, Japan).

SS-OCT scans were acquired through dilated pupils at the same day time and by an experienced technician. A macular 6 × 6 mm three-dimensional (3D) cube centered on the fovea was analysed three times, but only the best examination was selected for the analysis. Scans with low quality (<40/100), motion artifacts, or decentration were discarded. The choroidal segmentation was automatically performed using the on-board device software. In case of segmentation errors, manual corrections of individual *A*-scans were performed to fit the choroidal boundaries (from the outer edge of the hyper-reflective retinal pigment epithelial line to the inner edge of the sclera).

In every OCT image, the ETDRS grid was centered on the fovea, and measurements of the nine choroidal subfields were obtained and compared between groups.

Besides the classic ETDRS grid, a fovea-centered map composed of 30 × 30 cubes was generated with automatic measurements of CT. This map comprised 900 200 × 200 *μ*m cubes. Mean CT in every cube was exported and analysed. The left eyes were converted into the right eye format.

**Figure 1:**
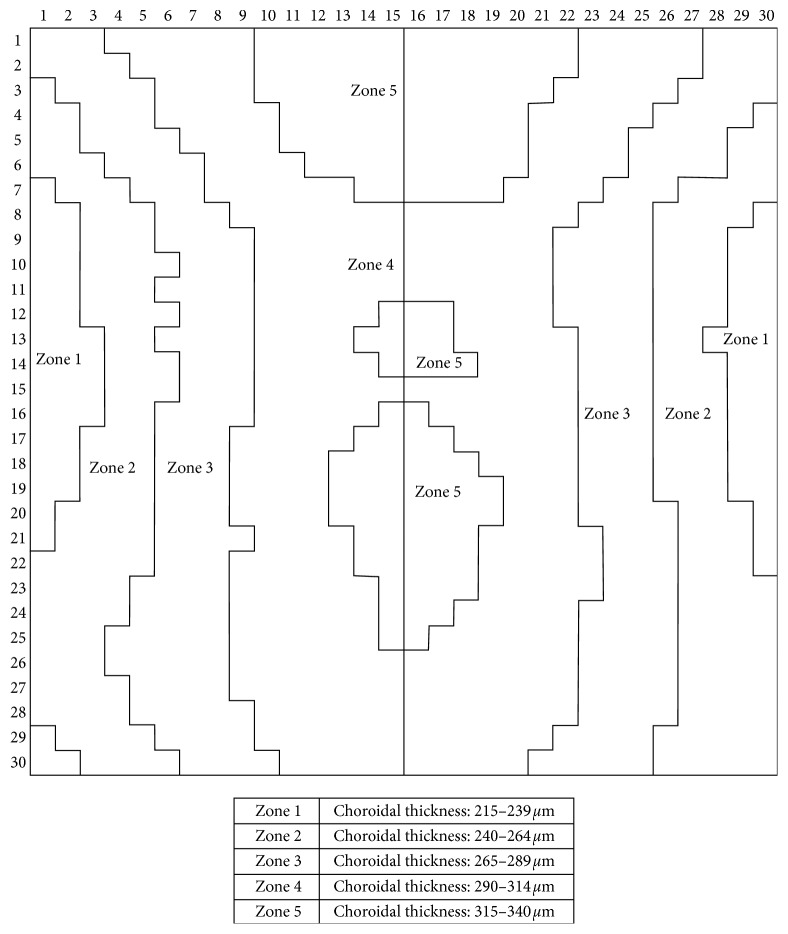
Choroidal zones in group 1 (young healthy individuals).

As a reference, young healthy subjects' choroid (group 1) was divided into different zones according to the mean CT in every macular cube (Figure 1). Zone 1 included those macular points with a CT between 215 and 239 *μ*m, zone 2 between 240 and 264 *μ*m, zone 3 between 265 and 290 *μ*m, zone 4 between 290 and 314 *μ*m, and zone 5 between 315 and 340 *μ*m. The 5 zones were then divided into nasal and temporal, obtaining a total of 10 measurements. The mean CT of equivalent zones was calculated and compared between groups.

The two-dimensional (2D) maps of the four study groups were created with Microsoft Word (Microsoft Corporation), and Microsoft Excel (Microsoft Corporation) was used for the three-dimensional (3D) maps.

### 2.3. Statistical Analysis

Statistical analyses were performed using IBM SPSS (version 23.0; IBM Corporation, Somers, NY, USA) statistical software. All variables followed a normal distribution as verified by the Kolmogorov–Smirnov test. A two-tailed Student's *t*-test was used to compare CT between groups using both the classic ETDRS grid (9 regions) and the new choroidal distribution (10 regions). In case of comparisons involving group 3, a Mann–Whitney *U* test was performed due to the insufficient number of cases. For all analyses, *p* < 0.05 was considered as statistically significant.

### 2.4. Demographics

We enrolled 102 eyes of 51 healthy young subjects (group 1), 60 eyes of 30 healthy aged subjects (group 2), 24 eyes of 12 high-myopic patients (group 3), and 110 eyes of 55 aged patients with diabetes mellitus type 2 (group 4) with mild or moderate diabetic retinopathy and without macular oedema. Mean age outcomes in the four study groups are displayed in Table 1. There were no differences between mean ages of groups 1 (young healthy) and 3 (young myopic) (*p*=0.79) and between groups 2 (aged healthy) and 4 (aged diabetic) (*p*=0.09). There were no differences between the best-corrected visual acuity between groups 1 and 3 (*p*=0.97), but there were between groups 2 and 4 (*p* < 0.001). There were no differences regarding intraocular pressure between groups 1 and 3 (*p*=0.83), between 2 and 4 (*p*=0.14), and between 1 and 2 (*p*=0.08). There were differences in AL between groups 1 and 3 (*p* < 0.001), but there were not between groups 1 and 2 (*p*=0.33) and between groups 2 and 4 (*p*=0.17).

## 3. Results

### 3.1. Choroidal Measurements

Average CT values are displayed in Table 2. When evaluating choroid with the ETDRS grid, the thickest choroid was found in the inner temporal (317.65 ± 72.30 *μ*m), inner superior (240.35 ± 62.92 *μ*m), outer superior (247.78 ± 61.79 *μ*m), and inner superior (191.68 ± 76.31 *μ*m) sectors in groups 1, 2, 3, and 4, respectively. When evaluating choroid with the new choroidal division, the thickest choroid was found in zone 5 nasal (320.93 ± 67.90 *μ*m), zone 5 temporal (236.51 ± 60.98 *μ*m), zone 5 temporal (250.50 ± 56.96 *μ*m), and zone 5 temporal (189.76 ± 66.24 *μ*m) in groups 1, 2, 3, and 4, respectively.

Table 3 shows the CT comparison between groups using ETDRS and the new division. CT was always significantly thicker (*p* < 0.01) in group 1 (young healthy) than in groups 2 and 3. Group 2 (aged healthy) always showed to have a thicker choroid (*p* < 0.02) than group 4 (aged diabetic). *p* values are shown in Table 3 too.

**Figure 2:**
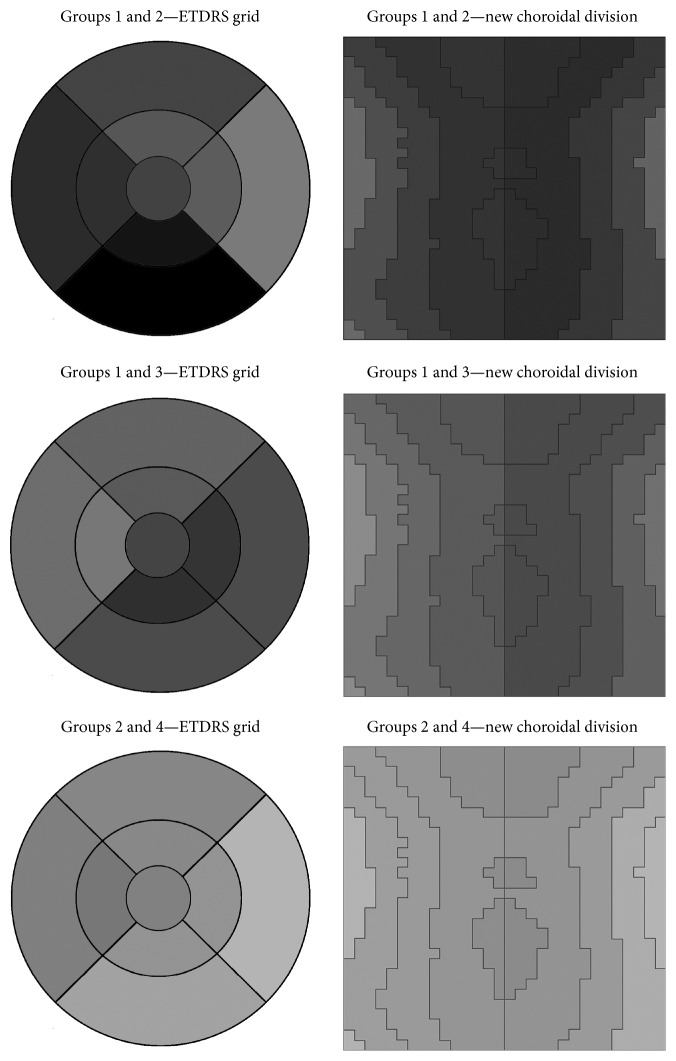
Mean choroidal thinning.

For a better understanding, Figure 2 shows a visual representation of the thinning using the ETDRS grid. In this figure, the darker, the more the choroid gets thinned.

### 3.2. Choroidal Maps Using the New Choroidal Division

**Figure 3:**
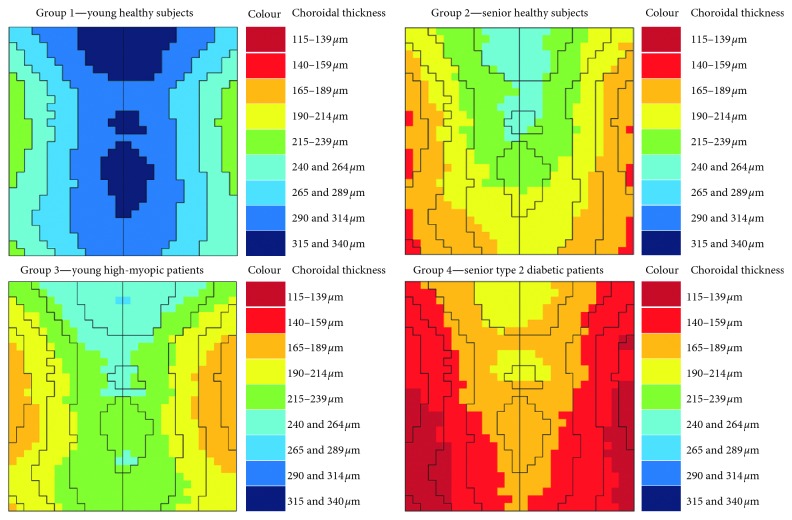
Two-dimensional representation of choroidal thickness.

**Figure 4:**
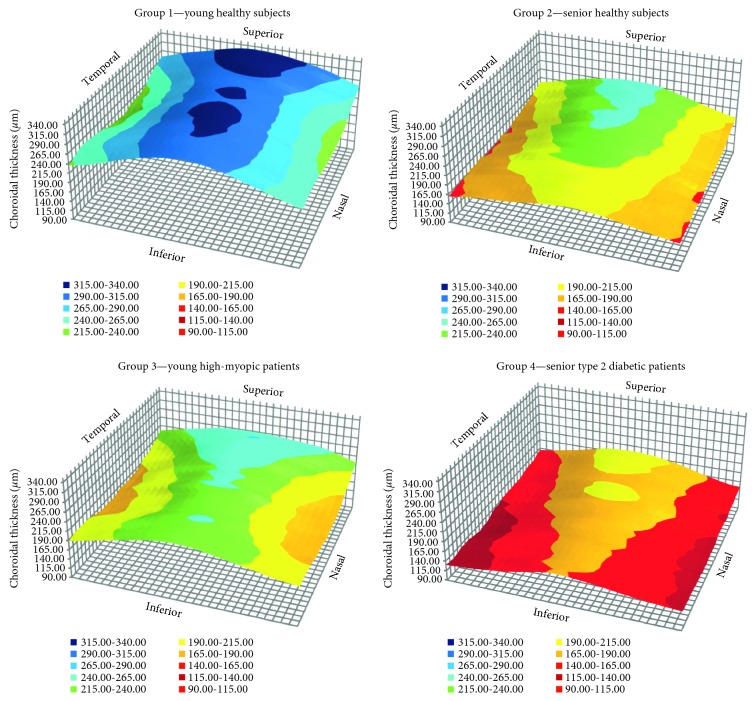
3D maps of choroidal thickness.

Figure 3 shows a colored 2D representation of CT in the four study groups. Black lines were drawn following the results in group 1 (young healthy) to allow an easier comparison.  Figure 4 shows a 3D representation of CT in the four study groups. It does not represent the real choroidal shape, as it is just a mathematical representation of its thickness on a flat surface. Nevertheless, the combination of these 2D and 3D maps allows better and easier understanding and visual comparison.

The thickest choroidal region was always superocentral to the fovea, showing a kind of ellipsoid shape. In young healthy individuals (group 1), it resembled a mountain range with its peaks and the valleys at both sides. In case of the other groups, the choroid tended to be flatter and the pattern was not always preserved. The aged healthy group (number 2) showed a higher reduction of CT in the inferior side, resulting in a choroidal pattern which resembled a single mountain peak. Something different happened with the young myopic patients (group 3), whose CT pattern stayed similar to the young healthy patients (group 1) but with a remarkable choroidal thinning on nasal and temporal sides. It resembled an inverted gorge. Finally, aged diabetic patients (group 4) showed to have the flattest choroid and its pattern was close to aged healthy patients' one (group 2), but instead of mountain peak, it was more similar to gathered hills.

## 4. Discussion

A thin choroid has been associated with ocular and systemic disorders, and sometimes can be useful in the differential diagnostic of some pathologies, such as between age-related macular degeneration and polypoidal choroidal vasculopathy [16]. One of the SS-OCT used so far for a deep comprehensive choroidal study has been Triton DRI. Its repeatability and reliability have been proved in healthy patients [18, 19] and in choroid-thickness thinning pathologies [20]. It gives similar measurements to the Zeiss Cirrus HD-OCT [21] (Carl Zeiss) although results should not be interchangeable [18]. It has been stated that automatic measurements reduce variability [22] although there is still little possibility of scan artifacts [23].

The most commonly used pattern is the ETDRS grid, as it is in retina, too. Nevertheless, the composition and functions of choroid has nothing to do with the ones of retina and its thickness does not follow the same pattern [5, 6, 12]. Although retinal thickness is not the same among ETDRS sectors, it does not differ too much [24], and that is why the ETDRS grid is an adequate useful pattern, whereas it might not be for choroid. However, no choroidal division has been proposed so far.

Choroid has usually been analysed with the ETDRS grid or with horizontal lines. At a first glance, the overall thickness map does not differ too much from the ones already published and mean CT values are similar, too. Some authors have described higher values of CT in superior parts [5, 6, 25] and the lowest in the outer macula area [5, 6, 26, 27], as well as we have. The fact of having analysed together right and left eyes should not have biased our study, as already stated by Chen et al. [28].

Shin et al. tried to make a choroidal map using radial OCT scans of the choroid and with the ETDRS grid, but they used a SD-OCT [26], and so the exact thickness values may differ. In our study, we found that the thickest choroid was always located in the superocentral area and the thinnest in temporal and nasal zones. With choroidal-thinning pathologies, the resulting CT map tends to be rather flat. However, the choroidal pattern differs depending on the pathology. Young healthy individuals show a mountain range pattern; aged healthy subjects, a mountain peak pattern; young high myopic, an inverted gorge pattern; and aged diabetic patients a gathered hills pattern, as displayed in Figures 3 and 4. This is why a 3D representation of CT has an importance, as it gives more information than ETDRS values alone.

The thickness range of 25 *μ*m for every color range in our maps is acceptable, as it is higher than the possible internal variation of the OCT but not so high that it remained unaltered with affecting pathologies. Rahman et al. stated that a manually measured change greater than 23 *μ*m in the subfoveal field may represent choroidal change when using SD-OCT with enhanced depth imaging (EDI) and manual measuring [22].

Although all the choroid and its sectors get significantly thinner with age, not all the sectors become equally affected. Bafiq et al. studied CT variation with age using manual measurements, and they found that the central choroidal thickness increased with age, the most thinned sector was the nasal outer one, and the second most thinned was the inferior ones, what differs to some extent to our results [11]. The outer inferior ETDRS grid sector is the most thinned, and the outer nasal one is the less thinned. As the latter has already been described as the thinnest choroidal zone even in healthy subjects, it is easy to understand that it is more difficult to achieve an even higher thinning.

On the contrary, high-myopic patients' choroid does not experience the same kind of thinning as with age. Their most thinned ETDRS sectors are the inferior and the nasal. Zhang et al. already found that the temporal choroid becomes less thinned than nasal, but they did not examine the superior or inferior choroid [12].

Finally, aged patients with mild and moderate DR suffer from a choroidal thinning which is softer in the outer nasal ETDRS sector. Their outer inferior ETDRS sector is affected from little thinning as well, but we should consider that this sector was already thinned because of age and then an even higher choroidal thinning would be rather difficult to achieve.

Thus, it is noticeable that, under these pathologies, there is a considerable flattening, and in those zones where CT was higher, choroidal thinning is also greater.

The strengths of this study include study-naïve patients and SS-OCT automatic measurements performed at the same day time. Furthermore, thickness maps seem easier to evaluate the choroid than manual measurements or ETDRS numerical values.

The main limitations of this study are a low number of young high-myopic patients and the small quantity of choroidal-thinning pathologies evaluated. It would be of interest how the whole macular choroid changes in other situations.

In conclusion, choroidal-thinning pathologies affect choroidal regions in a different way. This fact gains relevance especially in choroidal measurements because not all regions are interchangeable and CT should be measured in the proper place depending on the particular pathology. Therefore, lineal OCT examinations are inadequate for choroidal evaluation as superior and inferior macular regions remain unanalyzed. Second, choroidal thinning follows the following rule: the thicker the zone is in healthy subjects, the thinner it becomes when affected by any pathology. Third, 3D representations of CT provide us with visual information which helps us to make an easier and faster general valuation. In general lines, the choroid in young healthy individuals follows a mountain range pattern; in aged healthy subjects, it follows a mountain peak pattern; in young high-myopic patients, it follows an inverted gorge pattern; and in aged diabetic patients, it follows a gathered hills pattern. All these pathologies tend to make a flat uniform choroid.

## Figures and Tables

**Table 1:** Demographic and general ophthalmological factors.

	Group 1 (young healthy)	Group 2 (senior healthy)	Group 3 (young high myopic)	Group 4 (senior T2 DM)
Age (years)	27.31 ± 3.95	66.41 ± 7.54	27.69 ± 3.89	66.48 ± 7.59
BCVA (decimal scale)	0.99 ± 0.07	0.89 ± 0.13	0.99 ± 0.03	0.75 ± 0.23
IOP (mmHg)	16.07 ± 2.38	16.09 ± 2.56	16.81 ± 3.01	16.76 ± 2.96
AL (mm)	23.68 ± 0.73	23.97 ± 1.39	25.83 ± 0.70	23.21 ± 0.92
Number of eyes (patients)	102 (51)	60 (30)	24 (12)	110 (55)

BCVA = best-corrected visual acuity; IOP = intraocular pressure; AL = axial length.

**Table 2:** Choroidal thickness using the ETDRS grid.

ETDRS sector	Young healthy (group 1)		Aged healthy (group 2)		Young myopic (group 3)		Aged diabetic (group 4)	
	Mean (*μ*m)	SD	Mean (*μ*m)	SD	Mean (*μ*m)	SD	Mean (*μ*m)	SD
Center	315.73	37.12	237.75	68.57	239.00	60.30	186.24	69.89
Inner temporal	317.65	72.30	231.30	62.62	246.10	60.85	175.73	67.17
Inner superior	309.11	68.58	240.35	62.92	240.79	60.04	191.68	76.31
Inner nasal	285.95	70.71	218.63	70.19	201.57	59.60	173.82	75.09
Inner inferior	317.22	73.91	219.31	65.44	231.03	62.13	174.61	68.70
Outer temporal	300.63	70.89	212.80	56.27	240.70	58.47	160.24	59.38
Outer superior	312.02	67.68	235.46	63.92	247.78	61.79	185.55	68.34
Outer nasal	224.53	68.67	169.29	70.70	150.73	51.19	137.06	71.78
Outer inferior	302.42	74.12	199.13	62.81	228.59	56.20	160.28	63.21

*Sector of the new division*								
Zone 1 nasal	234.71	93.06	169.33	73.10	175.31	77.57	137.57	71.43
Zone 1 temporal	232.19	80.77	170.32	66.48	184.43	79.29	137.93	62.21
Zone 2 nasal	255.33	87.33	178.05	66.33	189.50	69.62	142.66	64.57
Zone 2 temporal	253.88	75.41	181.00	63.64	196.54	70.98	141.28	59.14
Zone 3 nasal	279.29	70.04	196.30	62.86	206.77	64.19	154.60	64.39
Zone 3 temporal	277.77	71.42	198.19	62.05	214.47	62.26	155.73	60.61
Zone 4 nasal	304.85	71.60	217.00	59.71	229.41	57.96	172.11	64.49
Zone 4 temporal	303.68	66.63	218.29	60.37	235.26	56.23	174.23	62.90
Zone 5 nasal	320.93	67.90	234.10	59.93	246.21	58.39	185.62	66.61
Zone 5 temporal	320.92	67.37	236.51	60.98	250.50	56.96	189.76	66.24

**Table 3:** Choroidal thickness comparison between groups.

ETDRS region	Groups 1-2		Groups 1–3		Groups 2–4	
	CT reduction (*μ*m)	*p*	CT reduction (*μ*m)	*p*	CT reduction (*μ*m)	*p*
Center	77.98	<0.001	76.73	<0.001	51.52	<0.001
Inner temporal	86.36	<0.001	71.54	<0.001	55.57	<0.001
Inner superior	68.77	<0.001	68.32	<0.001	48.66	<0.001
Inner nasal	67.30	<0.001	84.38	<0.001	44.81	<0.001
Inner inferior	97.89	<0.001	86.19	<0.001	44.70	<0.001
Outer temporal	87.83	<0.001	52.93	<0.001	52.56	<0.001
Outer superior	76.58	<0.001	64.25	<0.001	49.91	<0.001
Outer nasal	55.25	<0.001	73.80	<0.001	32.22	<0.001
Outer inferior	103.28	<0.001	73.83	<0.001	38.85	<0.001

*Sector of the new division*						
Zone 1 nasal	65.38	<0.001	59.40	<0.001	31.76	<0.001
Zone 1 temporal	61.88	<0.001	47.76	<0.001	32.38	<0.001
Zone 2 nasal	77.27	<0.001	65.83	<0.001	35.39	<0.001
Zone 2 temporal	72.89	<0.001	57.34	<0.001	39.72	<0.001
Zone 3 nasal	82.99	<0.001	72.52	<0.001	41.69	<0.001
Zone 3 temporal	79.57	<0.001	63.29	<0.001	42.47	<0.001
Zone 4 nasal	87.85	<0.001	75.44	<0.001	44.89	<0.001
Zone 4 temporal	85.39	<0.001	68.42	<0.001	44.05	<0.001
Zone 5 nasal	86.83	<0.001	74.71	<0.001	48.48	<0.001
Zone 5 temporal	84.40	<0.001	70.42	<0.001	46.76	<0.001

## Data Availability

All data of this study has been collected and stored at the Miguel Servet University Hospital in Zaragoza, Spain.

## References

[1] Ruiz-Medrano J., Flores-Moreno I., Peña-García P., Montero J. A., Duker J. S., Ruiz-Moreno J. M. (2014). Macular choroidal thickness profile in a healthy population measured by swept-source optical coherence tomography. *Investigative Opthalmology & Visual Science*.

[2] Ozdogan Erkul S., Kapran Z., Murat Uyar O. (2014). Quantitative analysis of subfoveal choroidal thickness using enhanced depth imaging optical coherence tomography in normal eyes. *International Ophthalmology*.

[3] Wakatsuki Y., Shinojima A., Kawamura A., Yuzawa M. (2015). Correlation of aging and segmental choroidal thickness measurement using swept source optical coherence tomography in healthy eyes. *PLoS One*.

[4] Esmaeelpour M., Považay B., Hermann B. (2010). Three-dimensional 1060-nm OCT: choroidal thickness maps in normal subjects and improved posterior segment visualization in cataract patients. *Investigative Opthalmology & Visual Science*.

[5] Hirata M., Tsujikawa A., Matsumoto A. (2011). Macular choroidal thickness and volume in normal subjects measured by swept-source optical coherence tomography. *Investigative Opthalmology & Visual Science*.

[6] Tan C. S. H., Cheong K. X., Lim L. W., Li K. Z. (2014). Topographic variation of choroidal and retinal thicknesses at the macula in healthy adults. *British Journal of Ophthalmology*.

[7] Tan C. S. H., Cheong K. X. (2014). Macular choroidal thicknesses in healthy adults-relationship with ocular and demographic factors. *Investigative Opthalmology & Visual Science*.

[8] Gabriel M., Esmaeelpour M., Shams-Mafi F. (2017). Mapping diurnal changes in choroidal, Haller’s and Sattler’s layer thickness using 3-dimensional 1060-nm optical coherence tomography. *Graefe’s Archive for Clinical and Experimental Ophthalmology*.

[9] Zhao M., Yang X. F., Jiao X. (2016). The diurnal variation pattern of choroidal thickness in macular region of young healthy female individuals using spectral domain optical coherence tomography. *International Journal of Ophthalmology*.

[10] Usui S., Ikuno Y., Akiba M. (2012). Circadian changes in subfoveal choroidal thickness and the relationship with circulatory factors in healthy subjects. *Investigative Opthalmology & Visual Science*.

[11] Bafiq R., Mathew R., Pearce E. (2015). Age, sex, and ethnic variations in inner and outer retinal and choroidal thickness on spectral-domain optical coherence tomography. *American Journal of Ophthalmology*.

[12] Zhang Q., Neitz M., Neitz J., Wang R. K. (2015). Geographic mapping of choroidal thickness in myopic eyes using 1050-nm spectral domain optical coherence tomography. *Journal of Innovative Optical Health Sciences*.

[13] Melancia D., Vicente A., Cunha J. P., Abegão Pinto L., Ferreira J. (2016). Diabetic choroidopathy: a review of the current literature. *Graefe’s Archive for Clinical and Experimental Ophthalmology*.

[14] Kase S., Endo H., Yokoi M. (2016). Choroidal thickness in diabetic retinopathy in relation to long-term systemic treatments for diabetes mellitus. *European Journal of Ophthalmology*.

[15] Galgauskas S., Laurinavičiūtė G., Norvydaitė D., Stech S., Ašoklis R. (2016). Changes in choroidal thickness and corneal parameters in diabetic eyes. *European Journal of Ophthalmology*.

[16] Dansingani K. K., Balaratnasingam C., Naysan J., Freund K. B. (2016). En face imaging of pachychoroid spectrum disorders with swept-source optical coherence tomography. *Retina*.

[17] Waldstein S. M., Faatz H., Szimacsek M. (2015). Comparison of penetration depth in choroidal imaging using swept source vs spectral domain optical coherence tomography. *Eye*.

[18] Matsuo Y., Sakamoto T., Yamashita T., Tomita M., Shirasawa M., Terasaki H. (2013). Comparisons of choroidal thickness of normal eyes obtained by two different spectral-domain OCT instruments and one swept-source OCT instrument. *Investigative Opthalmology & Visual Science*.

[19] Lee S. Y., Bae H. W., Kwon H. J., Seong G. J., Kim C. Y. (2016). Repeatability and agreement of swept source and spectral domain optical coherence tomography evaluations of thickness sectors in normal eyes. *Journal of Glaucoma*.

[20] Min J. K., Lee S., Kim J. S., Woo J. M., Yang H. S. (2017). Effects of diabetic macular edema on repeatability of retinal nerve fiber layer thickness measurements at the macular and peripapillary area using swept-source optical coherence tomography. *Current Eye Research*.

[21] Tan C. S. H., Ngo W. K., Cheong K. X. (2015). Comparison of choroidal thicknesses using swept source and spectral domain optical coherence tomography in diseased and normal eyes. *British Journal of Ophthalmology*.

[22] Rahman W., Chen F. K., Yeoh J., Patel P., Tufail A., Da Cruz L. (2011). Repeatability of manual subfoveal choroidal thickness measurements in healthy subjects using the technique of enhanced depth imaging optical coherence tomography. *Investigative Opthalmology & Visual Science*.

[23] Mansouri K., Medeiros F. A., Tatham A. J., Marchase N., Weinreb R. N. (2014). Evaluation of retinal and choroidal thickness by swept-source optical coherence tomography: repeatability and assessment of artifacts. *American Journal of Ophthalmology*.

[24] Wang J., Gao X., Huang W. (2015). Swept-source optical coherence tomography imaging of macular retinal and choroidal structures in healthy eyes. *BMC Ophthalmology*.

[25] Ouyang Y., Heussen F. M., Mokwa N. (2011). Spatial distribution of posterior pole choroidal thickness by spectral domain optical coherence tomography. *Investigative Opthalmology & Visual Science*.

[26] Shin J. W., Shin Y. U., Lee B. R. (2012). Choroidal thickness and volume mapping by a six radial scan protocol on spectral-domain optical coherence tomography. *Ophthalmology*.

[27] Manjunath V., Taha M., Fujimoto J. G., Duker J. S. (2010). Choroidal thickness in normal eyes measured using cirrus HD optical coherence tomography. *American Journal of Ophthalmology*.

[28] Chen F. K., Yeoh J., Rahman W., Patel P. J., Tufail A., Da Cruz L. (2012). Topographic variation and interocular symmetry of macular choroidal thickness using enhanced depth imaging optical coherence tomography. *Investigative Opthalmology & Visual Science*.

